# A physician-physiotherapist collaborative model in a family medicine teaching clinic

**Published:** 2018-11-12

**Authors:** Simon Deslauriers, Marie-Eve Toutant, Caroline Laberge, Annie St-Pierre, François Desmeules, Kadija Perreault

**Affiliations:** 1 Center for Interdisciplinary Research in Rehabilitation and Social Integration (CIRRIS), Centre intégré universitaire en santé et services sociaux de la Capitale-Nationale (CIUSSS-CN) Québec, Canada; 2 Faculty of Medicine, Université Laval, Québec, Canada; 3 Laurier Family Medicine Unit, CIUSSS-CN, Québec, Canada; 4 School of Rehabilitation, Faculty of Medicine, Université de Montréal, Québec, Canada; 5 Maisonneuve-Rosemont Hospital Research Centre, Québec, Canada

## Abstract

Persons with musculoskeletal disorders frequently seek care in family medicine clinics. However, musculoskeletal education provided in medical schools is often considered insufficient. The implementation of a collaborative model that integrates physiotherapists into teaching clinics may benefit the musculoskeletal training of medical residents. This paper describes a model developed in a family medicine teaching clinic by examining the interprofessional educational and collaborative activities implemented in this model. The model allowed to provide physiotherapy services, involve the physiotherapist in the training of family medicine residents and enhance interprofessional collaboration, particularly for the management of persons with musculoskeletal disorders.

## Abstract (fr)

*Les personnes ayant des troubles musculosquelettiques consultent fréquemment en cliniques de médecine de famille. Cependant, l’enseignement musculosquelettique dispensé dans les programmes de médecine est souvent considéré comme insuffisant. L’implantation d’un modèle de collaboration qui intègre les physiothérapeutes aux cliniques d’enseignement pourrait améliorer la formation des médecins résidents. Cet article décrit un modèle développé dans une clinique d’enseignement en médecine familiale en examinant les activités interprofessionnelles d’éducation et de collaboration implantées dans ce modèle. Le modèle a permis d’offrir des services de physiothérapie, d’impliquer le physiothérapeute dans la formation des médecins résidents et d’améliorer la collaboration interprofessionnelle, particulièrement pour la prise en charge des personnes ayant des troubles musculosquelettiques*.

## Introduction

Musculoskeletal disorders (MSDs) are one of the most frequent reasons for primary care consultations in Canada.^1,2^ Despite the predominance of MSDs in primary care settings, physicians and medical students reportedly have a low level of knowledge and clinical confidence in the management of MSDs.^3-5^ This could be partially explained by the insufficient musculoskeletal education in the curriculum of medical schools.^5-8^ Physiotherapists may help improve the musculoskeletal training of medical residents, as they are frequently involved in the management of MSDs and have extensive knowledge and competency in this field.^9^

This paper describes a collaborative model in which physiotherapists are integrated into a primary care team in a family medicine teaching clinic. We examined interprofessional educational and collaborative activities implemented in this model using clinical and administrative data. The ethics review board of the *CIUSSS de la Capitale-Nationale* approved this study as program evaluation.

## A collaborative initiative

In 2009, a physiotherapist was hired to join a primary care team in a university-affiliated Family Medicine Group (U-FMG) located in Québec City, Canada. The primary care team included approximately twenty-eight medical residents, seventeen family physicians, one physiotherapist as well as nurses, social workers, and nutritionists. This initiative aimed to 1) provide physiotherapy services, 2) involve the physiotherapist in the training of family medicine residents, and 3) enhance interprofessional collaboration.

### Interprofessional collaborative activities

**Figure 1:**
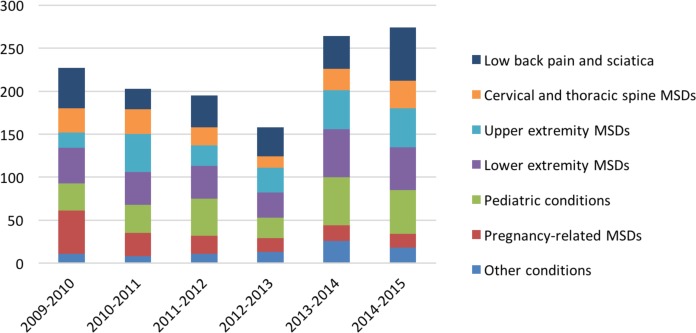
Number of physiotherapy referrals per reason of referral, per year.

Collaboration between physiotherapists and family medicine residents/physicians first took the form of medical referral to physiotherapy. A total of 1,321 referrals were made between 2009 and 2015, for an average of 220.2 ± 44.0 (mean ± standard deviation) per year (Figure 1). Moreover, on-site physiotherapists were able to provide prompt answers to oral requests made by family medicine residents/physicians. For example, they offered clinical advice or conducted joint assessments of particular patients with the residents/physicians. From 2013 to 2016, 468 patient-related informal discussions occurred between the physiotherapists and the residents and 452 between the physiotherapists and the family physicians, for a total average of 306.7 ± 73.1 discussions per year.

### Interprofessional educational activities

Physiotherapists contributed to the training of residents by providing 8.9 ± 4.5 planned teaching lessons (e.g., lectures and practice sessions on the musculoskeletal physical exam) and 36.0 ± 17.0 observation sessions per year. These observation sessions allowed residents to spend a few hours with the physiotherapist to gain insight into their clinical practices. Physiotherapists also took part in a weekly formal interprofessional meeting, during which a resident discussed a specific patient’s case with an interprofessional group.

## Conclusion

This innovative collaborative model in which physiotherapists are integrated in a family medicine teaching clinic encompasses a variety of interprofessional activities. Based on our experience, such an innovative model has the potential to enhance the musculoskeletal training of medical residents and the management of patients with MSDs in primary care. Consequently, we believe that this model has implications for researchers, decision makers and clinicians. Identifying barriers and enablers to the implementation of this model and assessing its efficacy could serve as suggestions for future research.
